# Patient-related outcome measures in medical education research 

**DOI:** 10.3205/zma001797

**Published:** 2026-01-15

**Authors:** Marjo Wijnen-Meijer, John Norcini

**Affiliations:** 1 Technische Universität Dresden, Medizinische Fakultät Carl Gustav Carus, Institut für Didaktik und Lehrforschung in der Medizin, Dresden, Germany; 2 Foundation for Advancement of International, Medical Education and Research, Philadelphia, USA

**Keywords:** medical education research, patient-related outcomes, patient-reported outcome measures (PROMs)

## Abstract

The use of patient-related outcomes in medical education research has gained traction over the past 25 years, yet it remains underutilized. In 2001, less than 7% of medical education publications included patient outcomes, despite the goal being the production of high-quality healthcare providers. This commentary discusses common sources of patient outcome data, their applications, and challenges. Administrative databases and Patient-Reported Outcome Measures (PROMs) are key sources of data, with PROMs capturing patients’ direct reports of their health status. PROMs are particularly useful when administrative data are scarce, such as in Europe. They can be employed to assess a variety of educational impacts, including the effect of physician experience on patient satisfaction and outcomes, as well as error rates in diagnosis and treatment.

Challenges in using such data include difficulty identifying appropriate outcomes and attribution of results to individual providers given patient-specific factors and the growing importance of team-based care. Consequently, large numbers of patients are needed to produce meaningful results. Despite these challenges, PROMs hold promise for improving medical education by focusing on what is most important – outcomes for patients.

## Abstract (de)

Die Verwendung von patientenbezogenen Ergebnissen in der medizinischen Bildungsforschung hat in den letzten 25 Jahren an Bedeutung gewonnen, wird jedoch weiterhin nur unzureichend genutzt. Im Jahr 2001 enthielten weniger als 7% der Publikationen in der medizinischen Bildungsforschung patient:innenbezogene Ergebnisse, obwohl das Ziel die Ausbildung von hochqualifizierten Gesundheitsdienstleistern ist. Dieser Kommentar erörtert häufige Quellen von Patient:innenergebnisdaten, deren Anwendungen und Herausforderungen. Verwaltungsdatenbanken und Patient-Reported Outcome Measures (PROMs) sind wichtige Datenquellen, wobei PROMs die direkten Berichte der Patien:innten über ihren Gesundheitszustand erfassen. PROMs sind besonders nützlich, wenn administrative Daten knapp sind, wie zum Beispiel in Europa. Sie können eingesetzt werden, um eine Vielzahl von Bildungswirkungen zu bewerten, einschließlich des Effekts der ärztlichen Erfahrung auf die Patient:innenzufriedenheit und Ergebnisse sowie der Fehlerquoten bei Diagnose und Behandlung.

Zu den Herausforderungen bei der Verwendung solcher Daten gehören die schwierige Identifizierung adäquater Ergebnisse und die Zuordnung der Ergebnisse zu einzelnen Anbietern angesichts patient:innenspezifischer Faktoren und der wachsenden Bedeutung der teambasierten Versorgung.

Infolgedessen sind große Patient:innenzahlen erforderlich, um aussagekräftige Ergebnisse zu erzielen. Trotz dieser Herausforderungen bieten PROMs vielversprechende Ansätze zur Verbesserung der medizinischen Ausbildung, indem sie den Fokus auf das legen, was am wichtigsten ist – die Ergebnisse für die Patient:innen.

## Introduction

In 2001, a review of 599 research papers in medical education journals found that less than 7% of the publications incorporated patient outcomes [1]. Given that the primary aim of medical education is to produce doctors who provide high quality care, this deficency was striking and it led to calls for a research agenda that evaluated educational interventions in light of their impact on health [2]. Over the past 25 years, this has created increasing interest in, and use of, clinical outcomes in educational research. In this commentary, we will describe the common sources of informaton about patient outcomes, offer examples of their potential applications in educational research and identify some of the challenges of using such data.

The most common source of information on patient outcomes is administrative databases. Although developed mainly for billing purpose, they now contain sufficient information to be the basis for judgments about quality of care. In some countries, this information is de-identifed and available for research purposes. In education, such data have been used to study areas such as the association of duty hours and licensure examination performance with patient outcomes [3] ,[4]. 

Alternatively, Patient-Reported Outcome Measures (PROMs) have become increasingly important in medical research and practice in recent years. PROMs are reports of a patient's health status that come directly from the patient and they may include symptoms, quality of life, functional status, and so on. PROMs are based on standardised questionnaires that collect data from patients. These instruments are designed to systematically capture and quantify patients' subjective and objective experiences.

## Examples

In the Europen context, large administrative databases are rarely available for research purposes. Hence, we will focus our examples on the potential uses of PROMs. 

### Comparison of patient satisfaction with treatment by trainees versus experienced physicians 

A comparison of patient satisfaction and treatment outcomes between patients treated by students/residents under supervision and those treated by experienced physicians illustrates the utility of PROMs. Specifically, studies in interprofessional training wards (ITW) have shown that patient satisfaction in this setting is higher than in conventional wards in terms of interaction and communication with the same quality of care [5]. While confidence in clinical decision-making skills is increased by participating in a training programme on an ITW, communication skills in particular do not appear to differ between students with and without experience on an ITW [6]. The article by Scheffer et al. also shows that patients are positive about treatment by students, under supervision. Patients appreciate the extra time students have for patients, as well as their way of communicating and their empathy [7].

### Comparison of error rate by trainees versus experienced physicians

Another area that illustrates the power of PROMs is an analysis of whether students and residents make more errors in the prescription of medication or diagnosis than experienced doctors, and importantly which errors these are. This provides useful information about any shortcomings in the curriculum. A good example is the study by Kalfsvel et al (2023), which investigated the effect of physician experience on the occurrence of prescribing errors [8].

### Exploring the effects of the introduction of a curriculum on patient-related outcomes (PROMs)

Another potential area of application is investigation of the effects of incorporating PROMs into the clinical decision-making curricula of medical students. As PROMs have not yet been widely used in this setting, it might be important and pioneering work. 

Unfortunately, examinations in medical education often focus on easily measureable facts and not clinical reasoning. Oral examinations also tend to concentrate on quickly verifiable aspects of a subject, while aspects of presentation, joint decision-making, and patient welfare are neglected. A possible study design could compare two groups of student in the final phase of medical school. One group would receive specific learning situations with the use of PROMs, the other group without this use. At the end of medical school the clinical decision-making skills of both groups could be evaluated.

### Involvement of patients in medical education

In addition to the integration of patient surveys, the scientific literature reflects the growing interest in the direct integration of patients and their assessments into medical training. Over the last ten years, an increasing number of publications have appeared on this topic. For example, a systematic review by Dijk et al. (2020) highlights the role of active patient involvement in undergraduate medical education [9]. Moreau et al. (2021) conducted an international survey that investigated patient involvement in medical education research and produced positive results [10]. A theoretical systematic review by Bennett-Weston et al. (2022) emphasised the importance of patient involvement in health and social care education [11].

### Challenges in using patient outcomes

Despite their attractiveness, the use of patient outcomes face at least three challenges [12]. First, research using these data has often focused on hospital-based care and/or procedures where the outcomes are relatively easy to define. However, large parts of practice occur in outpatient settings where chronic conditions predominate. In these settings, patient outcomes are more difficult to identify and they often unfold over relatively long periods of time. 

Second, patient outcomes are rarely the exclusive result of a health care provder’s intervention. The exact nature of the problems, as well as the severity of those illnesses, strongly influence the outcomes. Likewise, factor such as the patients’ resources as well as their ability and willingness to follow the care recommentions of the provider make a difference. Finally, care is typically provided in teams and this further diminshes the ability to attribute the results to individuals.

Third, most of the work on outcomes has included sizeable numbers of providers and patients. This is needed to adjust for factors other than the providers that influence the consequences of care and to generate enough power to draw reasonable conclusions. 

## Conclusion

The integration of Patient-Reported Outcome Measures (PROMs) into medical education offers a wide range of opportunities to improve the quality of training and patient care. PROMs make it possible to focus on the patient's perspective and thus improve the communication and decision-making skills of medical students. In addition, PROMs have the potential to directly involve patients and their opinions in medical training. Despite the limited data available to date, the growing number of research studies speaks to the interest and relevance of the topic.

## Authors’ ORCIDs

- Marjo Wijnen-Meijer: [0000-0001-8401-5047]
- John Norcini: [0000-0002-8464-4115]

## Competing interests

The authors declare that they have no competing interests. 
